# Valorisation of Recycled Cement Paste: Feasibility of a Short-Duration Carbonation Process

**DOI:** 10.3390/ma15176001

**Published:** 2022-08-30

**Authors:** André Silva, Rita Nogueira, Alexandre Bogas, João Abrantes, Dariusz Wawrzyńczak, Aleksandra Ściubidło, Izabela Majchrzak-Kucęba

**Affiliations:** 1 Civil Engineering Research Innovation Sustainability, Department of Civil Engineering, Instituto Superior Técnico, Universidade de Lisboa, Avenue Rovisco Pais, 1049-001 Lisboa, Portugal; 2 proMetheus, Instituto Politécnico de Viana do Castelo, 4900-347 Viana do Castelo, Portugal; 3 Department of Advanced Energy Technologies, Faculty of Infrastructure and Environment, Czestochowa University of Technology, Dabrowskiego 73, 42-201 Czestochowa, Poland

**Keywords:** carbon capture utilization and storage, short carbonation process, cement paste recycled powder, C-S-H carbonation, CO_2_ uptake

## Abstract

Cement paste powder (CPP) is a by-product of the recycling process of concrete with an elevated carbonation capability and potential to be recycled as a binding material in new concrete batches. The application of a carbonation treatment to CPP improves this potential even more, besides the evident gains in terms of CO_2_ net balance. However, the long duration usually adopted in this treatment, from 3 to 28 days, hampers the industrial viability of the process. We studied the feasibility of a short-duration carbonation process, with a duration of two hours, carrying out a comprehensive characterization of the material throughout the process. The test was performed on CPP with an average initial water content of 16.9%, exposed to a CO_2_ concentration of 80%. The results demonstrate two main carbonation rates: a rapid growth rate in the first 18 minutes of the process, involving all the calcium-bearing compounds in CPP, and a slow growth rate afterwards, where only C-S-H contributes to the carbonation reaction. During the 2 h carbonation process, the main CPP compounds, calcium silicate hydrate (C-S-H) and calcium hydroxide (CH), reached different carbonation degrees, 31% and 94%, with, however, close CO_2_ uptake values, 8% and 11%, respectively. Nevertheless, the total CO_2_ uptake for this process (≈19%) attained values not distant from the values usually obtained in a carbonation of 12 days or more (19–25%). Hence, these findings highlight the blocking role of C-S-H in the carbonation process, indicating that longer carbonation periods are only going to be useful if an effective carbonation of this compound is accomplished. In the present scenario, where CH is the main contributor to the reaction, the reduction in the process duration is feasible.

## 1. Introduction

The usefulness and versatility of concrete in the construction industry sector have made it the second most consumed material in the world by volume [1]. As a result of this consumption, concrete is responsible for an 5–8% of total CO_2_ emissions annually [2,3], which is expected to further grow, given the estimates of increasing concrete consumption by 2050. Furthermore, this high environmental impact of concrete is essentially attributed to the production of cement, its major component contributing to the carbon emissions (80–95 wt%). This condition is caused by the high CO_2_ emissions emitted during the cement production, i.e., 0.73–0.85 tonnes of CO_2_ by tonne of Portland cement assuming a minimum clinker content of 95% (OPC) [4,5,6].

In 2009, pursuing a reduction in carbon emissions by the cement industry, the Cement Technology Roadmap identified four levers with major potential for an effective carbon emission reduction in this industry [1]. Recent investigations have focused on two of the four outlined levers, namely, the carbon capture utilization and storage technology (CCUS) and the clinker substitution technology [7,8,9,10,11]. These studies explore the benefit from the carbonation potential of mature concrete at the end of its life cycle, and also from the potential reactivity of the carbonated material as a binding component in new concrete batches.

Even though the most common outlet for mature concrete at the end of its life cycle is still dumping and landfilling [12,13], studies concerning the conversion of mature concrete into recycled aggregates have increasingly developed, in an effort to promote a circular economy in the concrete industry [14,15]. During this recycling process, a fully hydrated cement paste powder (CPP) is obtained as a by-product, although with impurities, mainly crushed aggregate. This material has been applied in recent studies as a supplementary cementitious material (SCM) in mortars, with, however, poor performance results [16]. It was with the introduction of a CO_2_ curing process prior to the utilization of CPP, tapping into its carbonation potential, that a positive and effective increase in its performance was achieved. Lu et al. [8] reported that an incorporation of 10% and 20% of fully carbonated CPP in cement pastes produced an increase of 6% and 32% in compressive strength, respectively, when compared with the same incorporation percentages of uncarbonated CPP. Similarly, Mehdizadeh et al. [7] reported that an incorporation of 5–20% of fully carbonated CPP returned a positive increase in compressive strength even compared with a reference paste composed of 100% cement. However, the carbonation of CPP is still far from being an industrially viable process. Investigations specifically focused on this topic are still scarce, and those that focus primarily on the utilization of carbonated CPP as a SCM present a process with a long duration that hampers its viability, as shown in Table 1. In fact, carbonation processes usually take a long time, ranging between 12 and 28 days. The CO_2_ uptake is also far from the maximum theoretical capacity, which is close to 50% in Portland cement, as estimated through Steinour’s formula, further decreasing in CPP due to hydration to values that should reach ≈40% for a water/binder ratio of 0.45 [17,18,19]. Differently, Wu et al. [10] adopted a shorter-duration carbonation process of three days, at the expense of the carbonation degree, reporting a significant reduction in the carbonation efficiency.

CPP is mainly a hydrated cement paste composed of calcium silicate hydrate (C-S-H) and calcium hydroxide (CH), and also, in a lower percentage, by aluminate compounds, namely, ettringite (AFt) and monosulfate (AFm) aluminate, calcium carbonate ($C\bar{C}$), and remaining anhydrous cement particles [11,20,21]. Although C-S-H is considered as the primary phase of hydrated Portland cement that affects the properties of cementitious mixtures—namely, mechanical strength and durability—in the current context, its importance is mainly associated with its high CaO content. Naturally, after the carbonation process, CPP originates a cementitious material mainly composed of $C\bar{C}$ and C-S-H with a reduced C/S ratio, although the presence of silica gel (SH) can also be present for a sufficiently high value of CO_2_ uptake [11]. Moreover, the above-mentioned carbonation originates alterations in the C-S-H microstructure, as well as consumption of the CH, in a process that presents some similarities with the effect of aluminosilicate SCM, such as ashes or slags in cementitious materials [22]. Thus, besides the maximization of the CO_2_ uptake, the characterization of the carbonated products obtained from the process should also be persecuted, since they can affect the efficiency of the CPP to be used as a SCM and, consequently, the final performance of the CPP-added cement mixture. The evaluation of the CO_2_ uptake is currently computed from the $C\bar{C}$ quantification, usually by thermal analysis. Conversely, the chemical structure and composition of CPP during the carbonation process are more complex and based on the quantification of the CH, C-S-H, and other calcium-bearing components. The consumption of these components over time permits analyzing their contribution to the $C\bar{C}$ formation, further allowing the study of the carbonation reaction mechanisms in cementitious materials. More specifically, while CH reacts with CO_2_, originating $C\bar{C}$ and H_2_O, C-S-H reacts with CO_2_ differently. The carbonation reaction occurs with decalcification of the C-S-H structure, in which the CaO reacts with CO_2_, thus reducing the quantity of CaO in C-S-H and originating new connections between the tetrahedral silicate chains. Hence, to further analyze the carbonation reaction of C-S-H, the estimation of the C/S ratio provides an important decalcification indicator and, consequently, an estimation of its carbonation degree [23,24]. Similarly, the polymerization condition of the silicate tetrahedra can also provide an indication of the C-S-H carbonation degree [22,23]. Hence, the characterization of carbonation mechanisms throughout the process and the analysis of parameters influencing both CO_2_ uptake and CO_2_ emissions are fundamental in the industrial feasibility of carbonated CPP utilization.

**Table 1:** Carbonation process experiments reported in the literature (at 20 °C and for a CO_2_ pressure of 1 atm).

Carbonation Process				CO_2_ Uptake (%)	Ref.
Maximum Particle Size (μm)	CO_2_ Concentration (%)	Relative Humidity (RH) (%)	Duration (Days)		
75	99	60	-	24.3	[24]
75/150	20	65	28	21.2	[7]
100	20	70	12	19.4	[8]
75	20	65	28	23.9	[25]

In this study, we investigated the feasibility of a short-term (2 h) carbonation process of CPP, pursuing the effective use of CPP as a carbon store SCM, intending simultaneously to uncover the possible justification for longer carbonation processes applied by previous authors. To this end, the characterization of a generic short carbonation process was performed, with specific parameter settings, intending to analyze the CPP composition throughout the carbonation process. Qualitative and quantitative thermogravimetric analysis (TGA), X-ray diffraction (XRD), and nuclear magnetic resonance (NMR) were used to assess the structural and chemical characterization of the compounds obtained throughout the carbonation process.

## 2. Materials and Methods

### 2.1. Materials

Synthetic CPP was adopted in this work, obtained from laboratory-made cement pastes with controlled composition. This option was considered essential to accurately evaluate the CPP composition throughout the carbonation process, the main objective of this work, which is significantly impaired in the case of using the real highly variable CPP obtained from recycling facilities. Note that this is a common option in similar studies (mentioned in Table 1) and that the removal of the impurities from real CPP can be an option in the future. In this sense, Carriço et al. managed to retrieve CPP from concrete waste with nearly 90 vol % purity [26].

We used Portland cement (OPC)—CEM I 42.5R with a mineralogical composition of C_3_S = 56.7%, C_2_S = 16.6%, C_3_A = 10.0%, and C_4_AF = 9.2%; a specific surface (Blaine) of 452 m^2^/kg; and a density of 3070 kg/m^3^. Its chemical composition consists of CaO = 62.76%, SiO_2_ = 19.42%, Al_2_O_3_ = 5.39%, Fe_2_O_3_ = 3.00%, MgO = 1.74%, K_2_O = 0.53%, and Na_2_O = 0.12%. Lab-made CPP, with controlled composition and hydration degree, was produced from well-hydrated cement paste with a water/cement ratio of 0.45. After curing in a moist chamber for 1 year, the cement paste was crushed and ground using a jaw crusher, a roller mill, and a ball mill, followed by sieving through a 250 μm mesh. After this process, the water content of CPP was 1.5 wt%; prior to the carbonation process, this parameter was increased to 16.9 ± 0.5%, as water content is a known promoting condition of the carbonation reaction [27].

### 2.2. CPP Carbonation

**Figure 1:**
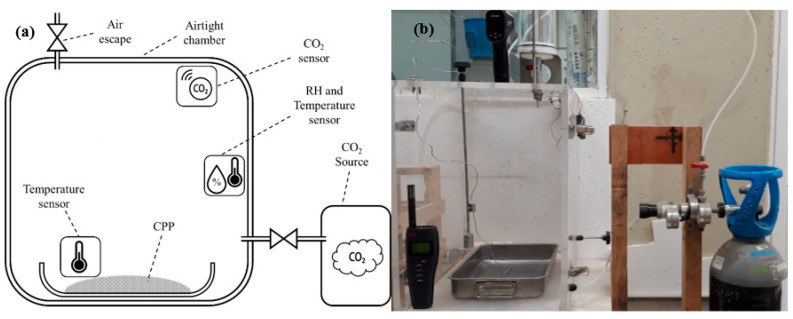
Schematic setup (**a**) and real setup (**b**) of the carbonation process equipment.

CPP carbonation was performed using an airtight chamber, with the schematic setup shown in Figure 1a and real setup in Figure 1b, and with an internal volume of ≈87 liters. In each carbonation test, 100 g of CPP with a thickness of 5 mm was exposed to a CO_2_ concentration of 80 vol% and an atmospheric pressure of 1 atm for 2 h, monitored by a Euro-GasMan CO_2_ sensor with an accuracy of ±70 ppm. The atmosphere’s relative humidity and temperature were settled at 60 ± 0.5% and 20 ± 0.2 °C at the beginning, respectively, and monitored over time using a Rotronic sensor. The temperature of CPP during the carbonation process was also monitored with Hanna temperature equipment with an accuracy of ±1.5 °C.

**Figure 2:**
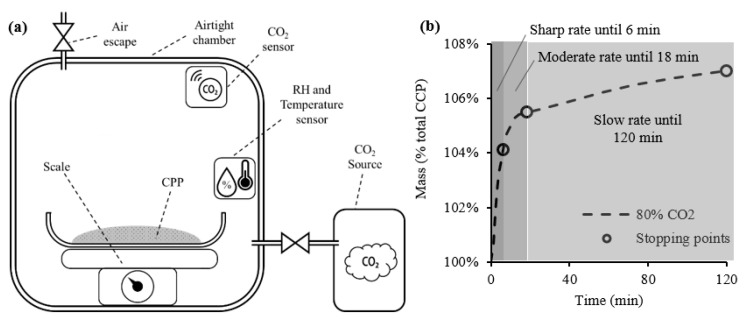
Equipment setup for the mass growth analysis (**a**) and stopping points for further CPP testing (**b**).

Two repetitions of the carbonation test were carried out, with shorter durations, to collect CPP samples over time for subsequent characterization. The carbonation period of these additional tests was defined considering the CPP mass growth rate during the 2 h test, using a similar setup, illustrated in Figure 2a. The CPP mass growth was recorded during the carbonation process by this setup (Figure 2b), and the corresponding derivative was computed to identify important transitions between growth rates. Hence, three different mass growth rates were identified (sharp, moderate, and slow), delimitated by the stopping points 1SP, 2SP, and 3SP shown in Figure 2b.

### 2.3. Test Methods

Three test methods were used in this investigation to analyze the CPP composition prior to and during the carbonation process, specifically at 1SP, 2SP, and 3SP. Thermogravimetric and X-ray diffraction analysis were used to determine the chemical composition of CPP during the carbonation process, also allowing us to estimate the C/S ratio of C-S-H and the carbonation degree of the CPP as a whole and of its main components individually. Nuclear magnetic resonance was adopted to analyze the chemical structure of C-S-H during the carbonation process.

#### 2.3.1. Thermogravimetric Analysis

Thermogravimetric analysis (TGA) was carried out on a NETZSCH STA 449F3 Thermobalance. Each sample, with around 60 mg, was heated at a rate of 10 °C/min in a He-gas flowing atmosphere, until a maximum temperature of 1000 °C. The temperature ranges of each mass loss were determined by the stepwise method. For a correct comparison between results, each sample was normalized to the non-ignited component using the loss on ignition value (LOI).

#### 2.3.2. Nuclear Magnetic Resonance

The ^29^Si magic angle spinning (MAS) spectra were obtained at 99 MHz by solid-state NMR using a Bruker Avance III 500 MHz NMR spectrometer, equipped with an HFX 4 mm Bruker MAS probe. Samples typically ∼150 mg were packed into a 4 mm zirconia rotor equipped with Kel-F caps. All samples were spun at 5 kHz. The ^29^Si MAS spectra were obtained by direct observation of ^29^Si with ^1^H decoupling and with a relaxation delay of 30 s. ^29^Si chemical shifts were referenced externally, with Tetrakis(trimethylsilyl)silane used as a standard (Si(CH_3_)_3_ at −9.3 ppm). The deconvolution software used was the OriginPro 9.0.0 SR2 b87 (Northampton, MA, USA).

#### 2.3.3. X-ray Diffraction

A conventional X-ray diffractometer was used (Bruker D8 Advance DaVinci, Karlsruhe, Germany) with Ni-filtered Cu-Kα radiation (λ = 1.5406 Å) produced at 40 kV and 40 mA, with a linear detector (Lynxeye 1-D) to perform XRD analysis. Datasets were recorded in the 5–80° 2θ range with a step size of 0.02°, 0.5 s per step, and a spin rate of 15 rpm. The Rietveld refinement was performed using TOPAS 5.0 (Bruker AXS, Karlsruhe, Germany) with the fundamental parameters approach used for phase quantification. The refinement of the crystal structure parameters, namely, atomic positions, site occupancies, and atomic displacement parameters, were not used, given the complex phase mixture present in cement, which can lead to erroneous results. Zinc oxide was used as an internal standard for the quantification of the amorphous phase. The results were also normalized to the non-ignited component.

The results from XRD were used to obtain the proportion of CaO to SiO_2_ (C/S) inside C-S-H and $C\bar{C}$ produced by the carbonation reaction of CH, C-S-H, and other residual calcium-bearing compounds. For this calculation, the chemical composition of the cement was also used, determined by X-ray fluorescence spectroscopy (XRF). The procedure is depicted in Equations (1)–(8), where E corresponds to the chemical oxide compounds of cement (e.g., CaO or SiO_2_), SP corresponds to the stopping point (from 0 to 3), and X corresponds to the anhydrous (e.g., C_2_S) and hydrate cement compounds (e.g., CH or C-S-H). X compounds were provided by XRD/Rietveld, although the amorphous ones are not discriminated but in the amorphous phase as a whole. The molar proportion (MR) of E in X is calculated by:

(1) $${\mathrm{MR}}_{E,X}=\frac{Z_{E,X}{\;\times \;m}_{E}}{{\;m}_{X}}\;(\%)$$

where $Z_{E,X}$ corresponds to the integer related to the stoichiometry of E in X, and $m_{E}$ and $m_{X}$ correspond to the molar mass of E and X, respectively. The quantity of E inside X at the stopping point SP is given by:

(2) $$E_{X,\;\mathrm{sp}}={\mathrm{MR}}_{E,X}\times X_{\mathrm{sp}}^{\mathrm{XRD}}\;(\%)$$

where $X_{\mathrm{sp}}^{\mathrm{XRD}}$ is the phase amount of the compound X at a given stopping point. Note that it is required to know the stoichiometry of the compound X to calculate ${\mathrm{MR}}_{E,X}$, which is possible for the compounds discriminated in the crystalline phase. Conversely, C-S-H has a variable stoichiometry and is not discriminated in the amorphous phase of XRD, precluding the calculation of $E_{C-S-H,\;\mathrm{sp}}$. However, the C/S ratio can be estimated in the simplified assumption that the amorphous phase is essentially composed of C-S-H, using the following equation:

(3) ratiospCS=CtotalXRF−∑XCX,spStotalXRF−∑XSX,sp

where $C_{\mathrm{total}}^{\mathrm{XRF}}$ and $S_{\mathrm{total}}^{\mathrm{XRF}}$ correspond to the CaO and SiO_2_ content obtained from XRF, respectively. $C_{X,\;\mathrm{sp}}$ and $S_{X,\;\mathrm{sp}}$ are the CaO and SiO_2_ content inside crystalline X compounds. Considering that the amorphous phase contains essentially C-S-H, but also AFm in a lower amount and eventual amorphous calcium carbonate (ACC), further efforts were made to improve the C/S estimate. Hence, the CaO content in AFm was estimated based on the Al_2_O_3_ content in the amorphous phase and on the stoichiometric proportion of CaO to Al_2_O_3_ (2.20) of known AFm compounds (namely, mono sulfoaluminates, calcium monocarboaluminate, and calcium hemicarboaluminate).

Regarding the $C\bar{C}$ produced by the carbonation reaction of CH and C-S-H, its calculation was performed assuming that the consumed CaO inside those compounds was entirely transformed into $C\bar{C}$. This calculation is particularly helpful in the estimation of the contribution of C-S-H to carbonation, given its undefined stoichiometry and, consequently, the lack of a precise generic carbonation reaction equation. The CaO reduction inside X between two consecutive stopping points, $∆C_{X,∆\mathrm{sp}}$, is obtained by:

(4) $$∆C_{X,∆\mathrm{sp}}=C_{X,\mathrm{sp}+1}-C_{X,\mathrm{sp}}\;(\%)$$

The $∆{C\bar{C}}_{X,∆\mathrm{sp}}$ is the (positive) variation of $C\bar{C}$ due to the carbonation of X in the same interval and is given by:

(5) $$∆{C\bar{C}}_{X,∆\mathrm{sp}}=∆C_{X,∆\mathrm{sp}}\frac{m_{C\bar{C}}}{m_{C}}\;(\%)$$

In Equation (5), ${\;m}_{C\bar{C}}$ and ${\;m}_{C}$ are the molar mass of $C\bar{C}$ and CaO, respectively. Thus, the $C\bar{C}$ obtained from the compound X at the stopping point SP is given by:

(6) $${C\bar{C}}_{X,\mathrm{sp}}={C\bar{C}}_{X,0}+\sum_{\mathrm{sp}}∆{C\bar{C}}_{X,∆\mathrm{sp}}\;(\%)$$

Finally, the carbonation degree of the compound X at the stopping point SP,${\;\mathrm{Cd}}_{X,\mathrm{sp}}$, is given by:

(7) $${\mathrm{Cd}}_{X,\mathrm{sp}}=\frac{{C\bar{C}}_{X,\mathrm{sp}}}{{C\bar{C}}_{X}^{\mathrm{Max}}}\;(\%)$$

where ${C\bar{C}}_{X}^{\mathrm{Max}}$ is the maximum theoretical $C\bar{C}$ obtained by the compound X and is given by:

(8) $${C\bar{C}}_{X}^{\mathrm{Max}}=C_{X,0}\frac{m_{C\bar{C}}}{m_{C}}\;(\%)$$

where $C_{X,0}$ is the CaO content of the compound X before carbonation.

## 3. Results and Discussion

### 3.1. CO_2_ Uptake and Water Content Variation

**Figure 3:**
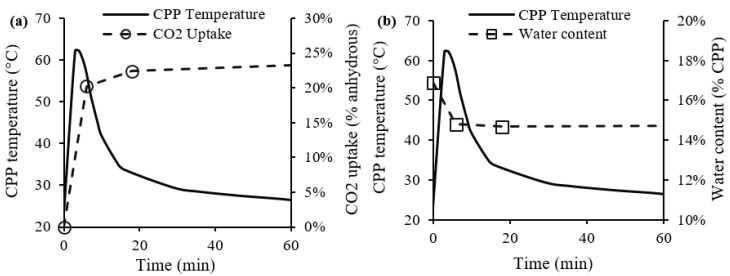
Results from carbonation process for 80% of CO_2_ concentration: temperature and CO_2_ uptake (**a**); temperature and water content (**b**).

Figure 3 shows the CPP temperature plotted with CO_2_ uptake (Figure 3a) and water content (Figure 3b) in the first hour of a 2 h generic carbonation process with a CO_2_ concentration of 80%. The results varied slightly for the remaining period (second hour). As observed in Figure 3a, in the first minutes of the process, there was a sharp increase in temperature with a simultaneous high rate of carbonation. In fact, in the first 6 min (1SP), the CO_2_ uptake reached 83% of the total value obtained by the end of the carbonation process. The water content reduced in the first minutes, and remained constant for the rest of the process (Figure 3b).

These results suggest that, provided some moisture in the CPP (16.9 ± 0.5%.), the 80% CO_2_ concentration promotes the diffusion and dissolution of CO_2_ into the porous water surrounding the CPP particles, following Equation (9). The contact between water and CPP particles supports the primary dissolution of CH into the porous water, releasing calcium ions, as indicated in Equation (10). The presence of calcium and carbonate ions in the pore solution leads to an exothermic reaction, expressed by Equation (11), which caused the burst of heat, as presented in Figure 3. The heat released promotes water evaporation from CPP [28], and water content decreases despite the water release in the reaction of Equation (11) [29,30]. The carbonation reaction continues, even though at a lower rate, absorbing more 17% of the total CO_2_ uptake after 1SP. As discussed later, this also involves the decomposition of other calcium-bearing compounds, such as C-S-H.

(9) $${\mathrm{CO}}_{2}\left(g\right)\to {\mathrm{CO}}_{2}\left(\mathrm{aq}\right)+H_{2}O\to H_{2}{\mathrm{CO}}_{3}\left(\mathrm{aq}\right)\to 2H^{+}\left(\mathrm{aq}\right)+{\mathrm{CO}}_{3}^{2-}\left(\mathrm{aq}\right)$$

(10) $$\mathrm{Ca}{\left(\mathrm{OH}\right)}_{2}\left(s\right)\to {\mathrm{Ca}}^{2+}\left(\mathrm{aq}\right)+2{\mathrm{OH}}^{-}\left(\mathrm{aq}\right)$$

(11) $${\mathrm{CO}}_{3}^{2-}\left(\mathrm{aq}\right)+{\mathrm{Ca}}^{2+}\left(\mathrm{aq}\right)+2{\mathrm{OH}}^{-}\left(\mathrm{aq}\right)+2H^{+}\left(\mathrm{aq}\right)\to {\mathrm{CaCO}}_{3}\left(s\right)+2H_{2}O+74\mathrm{kJ}/\mathrm{mol}$$

### 3.2. Carbonate Products from TGA

**Figure 4:**
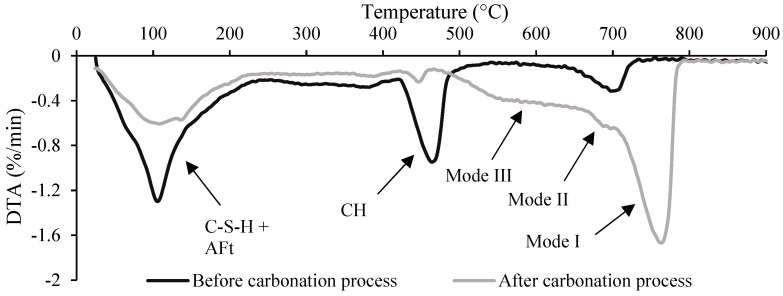
DTA results before and after the carbonation process.

Figure 4 shows the results from differential thermal analysis (DTA) obtained for uncarbonated CPP and 2 h carbonated CPP, illustrating the carbonation degree before and after the carbonation process. It is possible to identify three main peaks: from about 80 °C to 150 °C, related to the dehydration of C-S-H and calcium sulfate phases; from 400 °C to 500 °C, related to the dehydroxylation of CH; and from 500 °C to 800 °C, mainly related to the decarbonation of $C\bar{C}$ [31]. Three progressively increasing levels of carbonation can be distinguished in the latter peak [32,33,34,35,36]: Mode I is associated with a decomposition of well-crystalized $C\bar{C}$, calcite; Mode II is associated with the metastable phases of $C\bar{C}$, specifically aragonite and vaterite, which preferentially decompose in this mode; and Mode III is associated with amorphous $C\bar{C}$ (ACC). Thus, the TGA results suggest the simultaneous coexistence of different $C\bar{C}$ polymorphs, as reported by other studies on accelerated carbonation with similar experimental setups [34,37,38].

**Figure 5:**
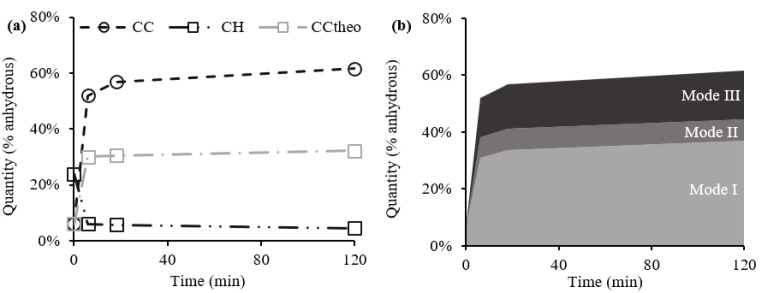
Results from $C\bar{C}$ and CH (**a**) and modes of $C\bar{C}$ (**b**).

Figure 5a shows the CH and $C\bar{C}$ contents obtained from TGA, as well as the theoretical $C\bar{C}$ obtained exclusively from CH, $C\bar{C}$_theo_, from the molar ratio between $C\bar{C}$ and CH. Note that the $C\bar{C}$ content of CPP is higher than the $C\bar{C}$ produced only from CH, indicating that other calcium-bearing compounds (C-S-H, AFt/AFm, and other minor compounds) should have also provided calcium ions for the carbonation reaction.

By the end of the carbonation process, there was still some residual CH (≈4.4%) in the carbonated CPP (Figure 4 and Figure 5a). A common explanation for this phenomenon is the formation of a $C\bar{C}$ coating around the CH particles, which slows down the CO_2_ access and the dissolution of CH necessary for the precipitation of $C\bar{C}$, expressed by Equation (10) [34,35,39]. Figure 5a also shows that there was a quick reaction of CH with CO_2_ in the first few minutes (until 1SP). After this quick consumption, the quantity of CH varied slightly throughout the carbonation process (only 1.6% from 1SP to 3SP). The high CO_2_ concentration inside the chamber and the reduction in water content inside CPP also supports a shorter migration distance of calcium ions away from the CH surface, favoring the precipitation of $C\bar{C}$ near the CH agglomerates [34,35,39]. Therefore, CH becomes less accessible, its carbonation rate is reduced, and some residual CH eventually remains uncarbonated.

Figure 5b presents a semi-quantitative estimation of the content of $C\bar{C}$ polymorphs in the sample, considering the different modes identified from DTA. A precise quantitative separation of the three modes is not possible, since temperature ranges are not completely distinctive. In accelerated carbonation processes with ACC production, the mass loss due to CO_2_ initiates at much lower temperatures, around 200 °C, overlapping the mass loss from CH dihydroxylation between 400 °C and 500 °C [33,34,35]. Moreover, ACC can be hydrated [40] and even arise intermingled with C-S-H which acted as precursor [41,42,43]. Thus, it is plausible to assume a temperature interval between ≈200 °C and ≈660 °C for Mode III, although occurring simultaneously with water release until around 500 °C. As for Modes II and I, temperature intervals are usually reported as [≈660 °C, ≈760 °C] and [≈760 °C, ≈950 °C], respectively [33,34,35]. In the present study, the following temperature intervals were considered from DTA derivative: [470 °C, 630 °C], [630 °C, 680 °C], and [680 °C, 1000 °C] for Modes III, II, and I, respectively. Figure 5b points out a higher content of Mode I ($C\bar{C}$ associated with well-crystalized $C\bar{C}$), followed by Mode III ($C\bar{C}$ associated with ACC), and finally, in a much lower amount, Mode II ($C\bar{C}$ associated with vaterite and/or aragonite). These intervals should be strictly considered as indicators of the main peaks of the products, keeping in mind that the corresponding weight losses may occur beyond the limits of the intervals. Therefore, the following discussion is developed assuming that the estimated values may contain some error. Nevertheless, these considerations suggest that the three modes arise as a consequence of carbonation. The comparison between Figure 5a,b also suggests that $C\bar{C}$ from Mode I is close to $C\bar{C}$_theo_ (from carbonation of CH).

The simultaneous presence of the $C\bar{C}$ polymorphs in OPC pastes subjected to accelerated carbonation is still debated, and different hypotheses are advanced. The most frequent explanation involves the calcium-bearing precursor: The well-crystallized $C\bar{C}$ from Mode I is attributed to the carbonation of portlandite, while the $C\bar{C}$ from Modes II and III is associated with the carbonation of C-S-H and hydrated calcium aluminate phases [37,44]. The C/S ratio of C-S-H has also been associated with different $C\bar{C}$ polymorphs [22,45]. Although the precise C/S intervals vary, both studies suggest that (i) for a low C/S ratio, aragonite is the main $C\bar{C}$ polymorph; (ii) for an intermediate C/S ratio, vaterite is the main compound; and (iii) for a high C/S ratio, simultaneous precipitation of calcite and vaterite occurs. ACC is reported to be present for every C/S; however, over time, it usually crystallizes into different and more stable $C\bar{C}$ polymorphs [45]. Other authors have linked the precipitation of calcite to a pH pore solution above 11, vaterite and aragonite to a pH below 11, and ACC to a pH below 9 [35,46]. Furthermore, Steiner et al. [27] also associated the $C\bar{C}$ polymorphs to the RH of the carbonation process, indicating that mainly calcite is formed for high RH values (91%), and vaterite, aragonite, and ACC are formed for lower RH values (57%). Nevertheless, it is important to point out that the crystallization pathway of calcium carbonate moves towards the most thermodynamically stable form: ACC, vaterite, aragonite, and, finally, calcite [27,47]. Although not all of these $C\bar{C}$ phases necessarily occur during the dissolution–reprecipitation process, the ambient conditions (e.g., pH-value) and precursor particularities (e.g., C/S ratio of C-S-H) affect the type of $C\bar{C}$ phases that occur, eventually leading to a subsequent transformation of a metastable $C\bar{C}$ polymorph (e.g., ACC) into a stabler $C\bar{C}$ (e.g., calcite) [35,37,47].

### 3.3. Crystalline Compounds Consumed and Produced from XRD

**Figure 6:**
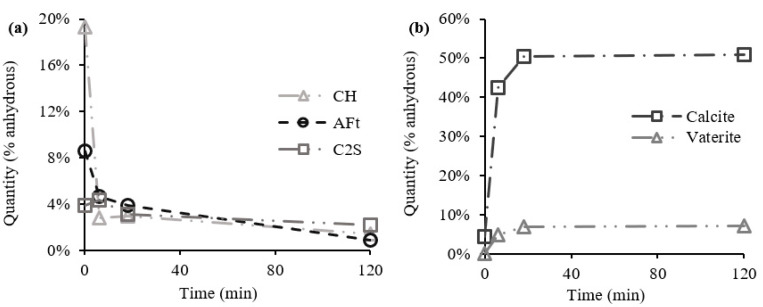
Crystalline phases consumed (**a**) and produced (**b**) from XRD.

The new arising compounds from the carbonation process were further analyzed from XRD with Rietveld refinement. Figure 6a,b presents the crystalline phases consumed and produced, respectively, during the carbonation process. In agreement with TGA, the results indicate that, besides CH, AFt and C2S also contributed with calcium ions to the carbonation reaction (Figure 6a). Similarly, these calcium-bearing compounds also decreased at a higher rate in the first six minutes of the carbonation process, and at a lower rate throughout the remaining duration. Moreover, other aluminate phases should be present in CPP, namely, calcium monosulfate (AFm), given the hydration degree of CPP. However, the reduced crystallinity of this compound prevented its detection in XRD [48,49].

Regarding the carbonate products, Figure 6b shows that a significant amount of $C\bar{C}$ precipitated into calcite. Vaterite was also detected, although to a lower extent, which confirms the existence of more than one polymorph of $C\bar{C}$ in carbonated CPP, as previously suggested by TGA, from the presence of different $C\bar{C}$ modes (namely, II and III). The presence of vaterite and the absence of aragonite suggest that $C\bar{C}$ from Mode II is essentially associated with the former compound (aragonite was not formed or was formed in a very low amount, below the detection range). Furthermore, the absence of aragonite in carbonated CPP can be evidence that silica gel was not formed during the carbonation process [37,45]. Note that both calcite and vaterite content are relatively stable after 2SP, presenting slight variations of 0.5% and 0.3%, respectively. However, in the same interval (2SP and 3SP), the CO_2_ uptake increases by 2.1% (Figure 3a), suggesting the presence of ACC in this range.

### 3.4. Cross Analysis between TGA and XRD

**Figure 7:**
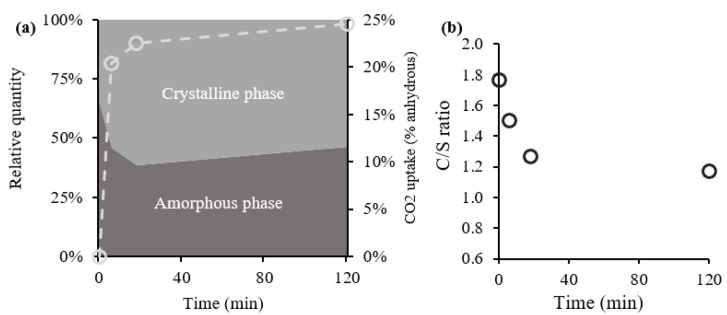
Comparison of amorphous and crystalline phases with the CO_2_ uptake in the dashed curve (**a**) and C/S ratio of C-S-H (**b**). C/S ratio computed from Equation (3).

The Rietveld refinement was used to evaluate the amount of the amorphous and crystalline phase in CPP, whose evolution along carbonation is shown in Figure 7a, together with the CO_2_ uptake obtained from TGA. Note that prior to the carbonation process, the amorphous phase should contain only hydrated amorphous compounds, mainly C-S-H and AFm. Then, as the carbonation reaction progresses, new calcium-bearing compounds with low crystallinity might arise, namely, ACC and other carbonated AFm phases (hemicarboaluminate and monocarboaluminate), besides the decalcified C-S-H [50]. Sevelsted et al. [23] reported that C-S-H carbonation originates a decalcified C-S-H with an amorphous structure until a limit C/S ratio of 0.67, along with $C\bar{C}$ and water, according to Equation (12). After this value, traces of an amorphous silicate hydrate phase start to appear, while $C\bar{C}$ and water are continuously produced. It is also pointed out that the $C\bar{C}$ obtained from C-S-H carbonation can have either an amorphous or crystalline structure, depending on the $C\bar{C}$ polymorph that precipitates, which in turn depends on the aforementioned parameters [23,51]. Hence, the main compounds affecting the amorphous phase amount in Figure 7a should be C-S-H, AFm phase, and ACC, while the crystalline phase is composed of the calcium-bearing compounds mentioned in Figure 6.

(12) $${\left(\mathrm{CaO}\right)}_{x+0.67}{\mathrm{SiO}}_{2}{\left(H_{2}O\right)}_{y}+{\mathrm{xCO}}_{2}\to {\left(\mathrm{CaO}\right)}_{0.67}{\mathrm{SiO}}_{2}{\left(H_{2}O\right)}_{y-z}+{\mathrm{xCaCO}}_{3}+{\mathrm{zH}}_{2}O$$

Figure 7a also points out a reduction in the amorphous content until 2SP at the expense of the crystalline phase, following the rapid CO_2_ uptake path that takes place in this stage. This result is the balance between the precipitation and dissolution contributions of both crystalline and amorphous phases. The quick and considerable precipitation of crystalline $C\bar{C}$ polymorphs calcite and vaterite (Figure 6b) yields a positive contribution to the crystalline content, with 99.1% and 96.5%, respectively, of the total $C\bar{C}$ produced occurring during this interval. Even though there is a carbonation of crystalline compounds (Figure 6a), this was compensated by the precipitation of crystalline $C\bar{C}$ achieved during this interval. Furthermore, the AFm phase is expected to follow a similar decrease rate to the AFt phase [52].

Additionally, the carbonation of C-S-H further contributed to the reduction in the amorphous content, as the C/S ratio of amorphous phase presented in Figure 7b demonstrates. This ratio was estimated as per Equation (3), considering the evolution of each crystalline phase and amorphous content by Rietveld analysis, and the chemical composition of cement (Section 2.1).

Note that the C/S ratio shown in Figure 7b refers to the C/S of the amorphous phase exclusively composed of eventual ACC and C-S-H and excluding AFm. In other words, this C/S is an indicator of the ratio inside C-S-H, overestimated in an amount proportional to the amount of ACC eventuality formed during the carbonation process. Nevertheless, the reduction trend of C/S in Figure 7b demonstrates that crystalline $C\bar{C}$ is forming, besides ACC. Moreover, considering the aforementioned thermodynamic instability of ACC and the high quantity of C-S-H in CPP, it is expected that the C/S ratio reflects the main composition of C-S-H. Therefore, Figure 7b shows that C-S-H is carbonating since the beginning, a progressive decalcification of this compound occurring. Up to 2SP, this phenomenon mainly involves the formation of crystalline $C\bar{C}$, because C/S is significantly reduced, which contributes to the further reduction in the amorphous content until 2SP. The eventual production of ACC from C-S-H since the beginning of the carbonation process is analyzed ahead.

After 2SP, as Figure 7a shows, there is an inversion of the amorphous decreasing trend in relation to the crystalline content. On one hand, Figure 6a shows that the carbonation of crystalline compounds occurs at a lower rate after 2SP, promoting a corresponding reduction in crystalline content. On the other hand, it is plausible to assume a progressive carbonation of crystalline aluminate phases (such as AFt and brazilianite) into amorphous products, as well as the formation of some ACC from the carbonation of C-S-H, according to Equation (12). The C/S ratio of C-S-H (Figure 7b) decreases at a lower rate after 2SP, promoting the reduction in the amorphous content, unless sufficient ACC precipitation occurs from C-S-H carbonation, which should be the case. As mentioned before, the stabilization of the precipitation of calcite and vaterite and the continuous increase in CO_2_ uptake further suggest the production of ACC in this interval. Moreover, the continuous reduction in C/S over the entire process confirms the formation of crystalline $C\bar{C}$ in Equation (12).

In other words, until 2SP, the consumption of CH and production of crystalline $C\bar{C}$ greatly increased the relative amount of the crystalline phase. Then, after the slowdown of CH consumption (2SP), carbonation of C-S-H (Equation (12)) and of calcium aluminates prevailed, increasing the relative amount of amorphous phases. The simultaneous presence of calcite and vaterite by the end of the carbonation process points towards a C-S-H with a C/S ratio higher than 1.02 [22]. Considering the C/S ratio of 1.17 (Figure 7b), a small overestimation of this ratio is plausible, caused by the ACC presence. Nevertheless, the estimated C/S ratio is still far from 0.67, suggested in Equation (12), which indicates that silica gel is not formed using the carbonation procedure considered in this study [49]. After 2SP, the significant reduction in the carbonation rate (Figure 5a) and the simultaneous stabilization trend of the C/S ratio (Figure 7b) suggest that carbonation of C-S-H tends to a limit, i.e., a longer period of carbonation has little influence. The NMR analysis performed ahead confirms the continuous but slower decalcification of C-S-H after 2SP.

**Figure 8:**
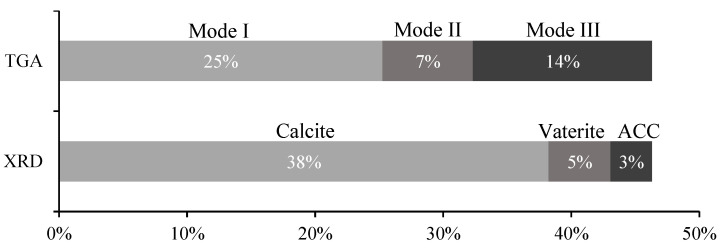
Comparison between TGA and XRD results regarding $C\bar{C}$ polymorphs produced at 1SP.

To further analyze the presence of ACC throughout the carbonation process, a comparison between the $C\bar{C}$ polymorph results obtained from TGA and XRD was plotted for the 1SP (Figure 8). The quantification of ACC in XRD bar was obtained by calculating the difference between the total $C\bar{C}$ content from TGA and the crystalline $C\bar{C}$ content. $C\bar{C}$ from each mode identified in TGA was obtained assuming the presence of some error, whose sources were previously depicted. Figure 8 enables us to further discuss this issue. According to this figure, the ACC content in 1SP is 3.2%, much lower than 14.0% of Mode III estimated by TGA. This overestimation suggests that $C\bar{C}$ from Mode III should also involve crystalline $C\bar{C}$ (calcite and vaterite). In accordance, the calcite amount by XRD is significantly higher than $C\bar{C}$ from Mode I (38% and 25%, respectively). Moreover, as previously mentioned, $C\bar{C}$ from Mode I almost matches the $C\bar{C}$ amount produced from CH (Figure 5), suggesting that $C\bar{C}$ with the highest crystallinity is obtained from this compound. Hence, Figure 8 also confirms the previous assumption that C-S-H originated crystalline $C\bar{C}$ (mainly calcite, but also vaterite) since the beginning of the carbonation process. Yet, this calcite from precursors other than CH starts decomposing at temperatures lower than those usually assumed for calcite produced from CH. Similar results were obtained in previous works, reporting that vaterite and ACC can be obtained from the carbonation of C-S-H and the calcium aluminate phase, justifying the presence of both these $C\bar{C}$ polymorphs as early as 1SP [37,44]. Nevertheless, it is expected that ACC progressively crystalizes into more stable $C\bar{C}$ polymorphs, yielding vaterite or calcite, depending on the characteristics of the chemical environment [27,45]. This consideration implies that further crystalline $C\bar{C}$ content produced after 1SP can also have the contribution of previously formed ACC besides the remaining calcium-bearing compounds. Finally, this result also confirms that the C/S ratio presented in Figure 7b is overestimated by the presence of ACC right from 1SP.

### 3.5. CO_2_ Uptake and Carbonation Degree of CH and C-S-H

**Figure 9:**
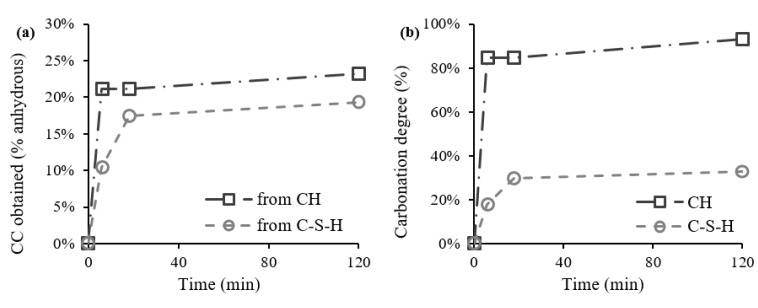
CC obtained from CH and C-S-H (**a**) and carbonation degree (**b**).

Considering the short duration of the accelerated carbonation process in relation to the common studies on this topic, the carbonation degree (Cd) achieved by the two major calcium-bearing compounds, CH and C-S-H, is discussed next. Figure 9a presents the $C\bar{C}$ obtained from CH and from C-S-H, according to Equation (6). Note that we adopted the simplified assumption that the amorphous phase is essentially composed of C-S-H. Their contribution is similar, especially considering that $C\bar{C}$ from C-S-H is underestimated due to the presence of ACC since the beginning. However, CH and C-S-H present a significantly different Cd, computed from Equation (7) and presented in Figure 9b. While CH presents a Cd of 93%, indicating almost total carbonation by the end of the process, C-S-H reveals a Cd of only 33%. This situation is a consequence of their different maximum theoretical $C\bar{C}$ contributions to the carbonation process: The estimated values are 59% for C-S-H (${C\bar{C}}_{\mathrm{CSH}}^{\mathrm{Max}}$) and 25% for CH (${C\bar{C}}_{\mathrm{CH}}^{\mathrm{Max}}$), calculated from Equation (8).

Furthermore, the $C\bar{C}$ obtained in 1SP from C-S-H (Figure 9a) is about 10%, which indicates that the maximum ACC content achievable in 1SP is 10%, if all $C\bar{C}$ obtained from Equation (12) is transformed into ACC. Therefore, it is confirmed that the 14% ACC content estimated from TGA is overestimated.

### 3.6. ^29^Si NMR Results

**Figure 10:**
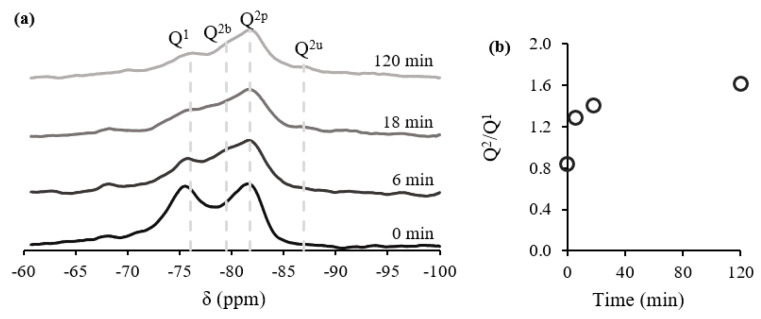
^29^Si NMR spectra (**a**), Q^2^/Q^1^ ratio (**b**).

^29^Si NMR was performed, aiming to further analyze the C-S-H chemical structure during the carbonation process. This technique is useful to investigate C-S-H alone, separated from the amorphous phase and, consequently, without the interference of ACC, unlike the C/S ratio obtained from XRD. Figure 10a shows the spectra of the ^29^Si NMR, exhibiting a first peak associated with the Q^1^ at ≈−76 ppm, a second peak associated with Q^2^ in the bridging position at ≈−78 ppm (Q^2b^), a third peak associated with Q^2^ in the pairing position at ≈−82 ppm (Q^2p^), and a fourth peak associated with Q^2^ in the bridging position at ≈−86 ppm (Q^2u^) [22,23,51,53]. The deconvolution of the NMR results enabled the calculation of the Q^2^/Q^1^ ratio, according to Equation (13), throughout the carbonation process (Figure 10b).

(13) $${\mathrm{ratio}\;Q}^{2}/Q^{1}=\frac{Q^{2b}+Q^{2p}+Q^{2u}}{Q^{1}}$$

The increase in Q^2^/Q^1^ throughout the process confirms that C-S-H provided calcium ions into the carbonation reaction since the beginning, in agreement with previous results, i.e., the C/S ratio variation in the amorphous phase (Figure 7b). This behavior indicates that C-S-H polymerization has a decreasing trend throughout the process, being particularly small after the 2SP, in agreement with the C/S ratio.

Furthermore, Q^3^ and Q^4^ sites were not observed, confirming that silica gel was not obtained in this short-duration carbonation process, opposed to what is reported in longer ones (Table 1). This is agreement with the absence of aragonite in XRD analysis.

## 4. Conclusions

We investigated the feasibility of a short-duration carbonation process to be applied to CPP. To this end, we characterized CPP throughout the carbonation process using different analysis techniques to investigate a generic two-hour carbonation process with a CO_2_ concentration of 80%. The tested carbonation conditions enabled a CO_2_ uptake of 24.6%, which, considering the CaO content in CPP, corresponds to a final carbonation degree of 47.9% in just two hours. Moreover, most of the carbonation was achieved in the first 18 min (CO_2_ uptake of 22.5%) of the carbonation process, and only 2.1% of CO_2_ uptake was achieved after this point.

Prior to the carbonation process, the CPP was essentially composed of C-S-H, CH, and calcium aluminate phases (AFm and AFt) in a lower amount. Carbonation of CPP originated a cementitious material mainly composed of calcium carbonate and decalcified C-S-H. The contribution of C-S-H and CH for the formation of $C\bar{C}$ is rather similar, yet the different CaO content of each compound indicates that CH is almost fully carbonated (93%), while C-S-H reveals a carbonation degree of 33%. The accelerated carbonation conditions provided in this process promoted the simultaneous formation of $C\bar{C}$ polymorphs (calcite, vaterite, and ACC) since the beginning of the process (less than 6 min). Furthermore, C-S-H revealed a polymerization and decalcification reaction throughout the carbonation process, demonstrating an initial higher reaction rate until the 18th minute and then a slow ongoing reaction. Nevertheless, the duration of this two-hour carbonation process was not sufficient for the formation of silica gel, as reported by other authors [47].

The temperature and water content of CPP showed high variation until the 18th minute, then stabilizing beyond this point, which confirmed the initial higher carbonation reactivity of CPP. In addition, the consumption of calcium-bearing cementitious compounds in CPP, namely, CH, C_2_S, and AFt, was also more substantial in this stage of the process, suggesting that the carbonation rate of C-S-H might be the rate controlling mechanism later on, ruling the CO_2_ uptake of CPP during the remaining part of the process.

These considerations point out two main phases of the carbonation process: rapid growth (until the 18th minute) and slow growth (between the 18th minute and 2nd hour). Two growth rates were identified within the first phase of the rapid carbonation growth: a sharp rate until the 6th minute, where almost all the CH is consumed with the corresponding production of $C\bar{C}$, and a more moderate rate, between the 6th and 18th minute, where a deceleration of CH consumption occurs. Table 2 summarizes the qualitative variation in the main phases of CCP during the carbonation process.

The investigation of this short-duration process revealed that the low carbonation rate of C-S-H during the second part of the process (slow growth) hindered a higher carbonation of CPP, suggesting that this is the main reason for the long-duration carbonation processes applied by other authors. Nevertheless, the conditions of this accelerated carbonation process enabled a significant carbonation of CPP during the rapid growth stage, with contributions from all the calcium-bearing compounds, including C-S-H. Therefore, this short-duration carbonation process showed promising results. Further work should be focused on a parametric analysis of some influencing factors on the carbonation process presented here, seeking the simultaneous maximization of the CO_2_ uptake and CPP performance. It would be also important to explore the transposition of this small-scale process into an industrial application, whose viability requires the adaptation of some of the current testing conditions, such as increasing the amount of CPP per carbonation process and reducing the CO_2_ concentration inside the chamber to the real values of flue gases.

## Figures and Tables

**Table 2:** Qualitative variation in the main phases of CPP during the carbonation process.

Carbonation Process	Time Period	CPP Phase Consumption			Carbonate Production		
		CH	C-S-H	Aluminate Phase	ACC	Vaterite	Calcite
**Rapid growth**	0–6 min (1SP)	++	++	++	+	+	++
	6–18 min (2SP)	0	++	+	+	+	+
**Slow growth**	18–120 min (3SP)	0	+	0	+	0	0

Legend: 0 constant; + significant; ++ very high.

## Data Availability

Data available on request due to restrictions.
